# Myocardin Family Members Drive Formation of Caveolae

**DOI:** 10.1371/journal.pone.0133931

**Published:** 2015-08-05

**Authors:** Katarzyna K. Krawczyk, Ingrid Yao Mattisson, Mari Ekman, Nikolay Oskolkov, Rebecka Grantinge, Dorota Kotowska, Björn Olde, Ola Hansson, Sebastian Albinsson, Joseph M. Miano, Catarina Rippe, Karl Swärd

**Affiliations:** 1 Department of Experimental Medical Science, BMC D12, Lund University, Lund, Sweden; 2 Department of Clinical Sciences, Lund University, Malmö, Sweden; 3 Department of Medicine, Aab Cardiovascular Research Institute, University of Rochester School of Medicine and Dentistry, Rochester, New York, United States of America; University of British Columbia, CANADA

## Abstract

Caveolae are membrane organelles that play roles in glucose and lipid metabolism and in vascular function. Formation of caveolae requires caveolins and cavins. The make-up of caveolae and their density is considered to reflect cell-specific transcriptional control mechanisms for caveolins and cavins, but knowledge regarding regulation of caveolae genes is incomplete. Myocardin (*MYOCD*) and its relative MRTF-A (*MKL1*) are transcriptional coactivators that control genes which promote smooth muscle differentiation. MRTF-A communicates changes in actin polymerization to nuclear gene transcription. Here we tested if myocardin family proteins control biogenesis of caveolae via activation of caveolin and cavin transcription. Using human coronary artery smooth muscle cells we found that jasplakinolide and latrunculin B (LatB), substances that promote and inhibit actin polymerization, increased and decreased protein levels of caveolins and cavins, respectively. The effect of LatB was associated with reduced mRNA levels for these genes and this was replicated by the MRTF inhibitor CCG-1423 which was non-additive with LatB. Overexpression of myocardin and MRTF-A caused 5-10-fold induction of caveolins whereas cavin-1 and cavin-2 were induced 2-3-fold. PACSIN2 also increased, establishing positive regulation of caveolae genes from three families. Full regulation of *CAV1* was retained in its proximal promoter. Knock down of the serum response factor (SRF), which mediates many of the effects of myocardin, decreased cavin-1 but increased caveolin-1 and -2 mRNAs. Viral transduction of myocardin increased the density of caveolae 5-fold *in vitro*. A decrease of CAV1 was observed concomitant with a decrease of the smooth muscle marker calponin in aortic aneurysms from mice (C57Bl/6) infused with angiotensin II. Human expression data disclosed correlations of *MYOCD* with *CAV1* in a majority of human tissues and in the heart, correlation with *MKL2* (MRTF-B) was observed. The myocardin family of transcriptional coactivators therefore drives formation of caveolae and this effect is largely independent of SRF.

## Introduction

Caveolae are omega-shaped pits in the plasma membrane with a diameter in the range of 50–100 nm that constitute a specialized lipid domain enriched in sphingolipids, cholesterol and phosphatidylinositol (4,5)-bisphosphate [1]. Caveolins, integral membrane proteins encoded by three distinct genes, are essential for the formation of caveolae [2]. Work performed over the last decade has identified additional proteins that contribute to the genesis, structure and trafficking of caveolae. Cavins, for example, are cytosolic proteins forming distinct sub-complexes that cover the cytosolic surface of caveolae [3, 4]. Genetic deletion of cavins leads to partial [5–7] or complete [8–10] loss of caveolae as does deletion of caveolins [11, 12]. Two other proteins, Pacsin2 and Ehd2, also play roles for the membrane curvature in caveolae [13] and for their membrane confinement [14]. As a result of overlapping functions within the caveolin and cavin families, caveolae with distinct protein compositions exist in different tissues [6, 15]. The composition of caveolae is thought to reflect cell-specific transcriptional control mechanisms for caveolins and cavins, and unraveling those mechanisms was recently identified as a major challenge in the field [16].

Caveolae are present in numerous tissues, including striated and smooth muscle [17, 18], endothelial cells [19] and adipocytes [20], where they are considered to influence signaling, trafficking and lipid metabolism. For caveolae to form, caveolin-1 or -3 and cavin-1 are required. In humans, null mutations in caveolin-3 (*CAV3*), which is expressed primarily in striated muscle, give rise to limb girdle muscular dystrophy [21]. Mutations in caveolin-1 (*CAV1*), on the other hand, result in congenital lipodystrophy [22]. Cavin-1 (*PTRF*) mutations give rise to a combined state of lipo- and muscular dystrophy and increase the risk of cardiac arrhythmias [23, 24]. A variety of other changes are present, including in smooth muscle, which probably reflects the contribution of cavin-1 to genesis of caveolae in all tissues. Rare congenital diseases thus underscore critical roles of caveolae in striated and smooth muscle and in adipocytes.

The myocardin family of proteins (MYOCD; MKL1/MRTF-A, MKL2/MRTF-B, MASTR) are transcriptional coactivators that bind to the serum response factor (SRF). This leads to transcription of a subset of SRF target genes that are important for myogenic differentiation and cytoskeletal organization [25, 26]. Binding of myocardin family proteins to SRF occurs in competition with ternary complex factors, such as ELK1, and the ELK1/SRF complex controls a distinct subset of genes that promote cell growth [27]. In this manner SRF influences different gene programs depending on the associated coactivator. Some myocardin family targets, and all MASTR targets, are controlled in an SRF-independent manner. An example is tenascin-C whose transcription is mediated by the SAP domain (from SAF-A and–B/Acinus/PIAS/) and occurs independently of SRF [28]. An important activation mechanism for two of the myocardin family coactivators (MRTF-A/MRTF-B) is altered actin polymerization; they bind to globular actin in the cytoplasm and translocate to the nucleus upon formation of actin filaments [29]. Genetic elimination of myocardin results in early embryonic lethality with severe vascular defects, halted vascular smooth muscle differentiation, and a hypoplastic heart [30–32]. Deletion of *MKL2*/MRTF-B results in a similarly serious, yet partly distinct, phenotype with abnormal arterial patterning and severe heart malformations [33]. *MKL1*/MRTF-A knockouts survive but cannot nurse their offspring due to impaired development of myoepithelial cells in the breast [34]. Essential roles of myocardin family coactivators have moreover been demonstrated in skeletal muscle [35], in endothelial cells [36] and in adipocytes [37]. Myocardin family proteins thus play pivotal roles in cell types that are characterized by high densities of membrane caveolae.

Important regulatory influences on the genes responsible for formation of caveolae have been identified [38–45], but no single mechanism has been identified that readily accounts for the tissue distribution of caveolae. The impetus for the present work was provided by the presence of SRF-binding sequences referred to as a CArG boxes upstream of cavin-1 gene (*PTRF*) and at the cavin-2 locus (*SDPR*) [46]. The former CArG box has been demonstrated by chromatin immunoprecipitation to be occupied by SRF and is characterized by high H3K27-acetylation (UCSC Genome Browser). We therefore addressed the hypothesis that cavin and caveolin expression is controlled by members of the myocardin family. Our studies unraveled forceful regulation of caveolin and cavin expression by myocardin and MRTF-A and overexpression of myocardin was found to increase the density of caveolae *in vitro*. A mouse aneurysm model was used to show *in vivo* co-regulation of the smooth muscle differentiation marker calponin with caveolin-1 in the setting of vascular injury. Using publicly accessible mRNA expression data we also found correlations between myocardin family members and caveolin-1 in numerous human tissues. These analyses argue that myocardin family proteins explain a sizeable, if not major, fraction of the naturally occurring variation of caveolin-1 expression.

## Materials and Methods

### Ethics statements

Human coronary artery smooth muscle cells were purchased from Gibco (Life Technologies) who ensures that written informed consent was obtained for use of tissue and its derivatives for research purposes (https://tools.lifetechnologies.com/content/sfs/COAPDFs/2012/1130140_C0175C.pdf). The mouse experimental protocol was approved by the Malmö/Lund Ethical Committee for animal experiments (the equivalent of an Institutional Animal Care and Use Committee; permit numbers M46-13 and M57-14), and animal handling conformed to national guidelines and the European Communities' Council Directive 86/609/EEC. Surgery was performed under isoflurane anaesthesia in sterile conditions, and all efforts were made to minimize suffering. The completed ARRIVE guidelines checklist is provided in in S1 Fig.

### Animals

In this study we used 10 weeks old male C57BL/6J mice (Taconic Biosciences, Denmark) weighing 20-25g. Mice were housed at the conventional animal facility at BMC, Lund, and maintained on a 12h light/dark cycle at room temperature. Health and microbiological status was monitored using FELASA guidelines and food was available *ad libitum*. A maximum of 6 mice were housed in open cages with aspen chip bedding. Every other mouse was assigned to angiotensin treatment and six mice in total received angiotensin treatment whereas six mice received saline. Blood pressures were recorded one cage at a time. For comparisons of tissue vs. smooth muscle cells in culture, three test naïve C57BL/6J mice were sacrificed and aortae were dissected. All measures were taken to reduce the number of animals used.

### Cell culture and adenoviral transduction

Human coronary artery smooth muscle cells (hCASMC, lot 1130140, Gibco, Life Technologies) were used at passages 3 to 8. Cells were cultured in Medium 231 (Life Technologies) with 5% smooth muscle growth supplement (“serum”, Life Technologies, M-231-500) and 50U/50μg/ml penicillin/streptomycin (Biochrom, A 2212). For polymerization of actin, cells were treated with 100 nM jasplakinolide (TOCRIS Bioscience, Bristol, UK, cat no. 2792) or a corresponding volume of DMSO (Sigma Aldrich, D5879) for 24h. In a subset of experiments, jasplakinolide-treated cells (24h) were further exposed to cycloheximide (1ug/ml, Sigma) for 36h and 48h. Latrunculin B, which was used for depolymerization of actin, is inactivated by serum. For treatment with this substance we therefore first starved cells in Medium 231 with 2% smooth muscle growth supplement (SMGS) 24h after seeding. After another 24h, 250 nM latrunculin B (Calbiochem, #76343-94-7), or the equivalent volume of DMSO (Sigma Aldrich, D5879), were added for 24h [47]. For dose-response relationships, cells were seeded and starved as described above and then treated with 10 nM, 50 nM, 100 nM, 300 nM and 1 μM of latrunculin B, or DMSO as vehicle, for 24h. hCASMC were transduced using 100 MOI (multiplicity of infection) of Ad-CMV-MYOCD-HA or 20 MOI of Ad-CMV-MRTF-A 24h after seeding. Cells were then maintained in virus-containing media for 96h. Ad-CMV-SMARCA2 (Vector Biolabs) was used at 100 MOI. Ad-CMV-null (20 or 100 MOI, Vector Biolabs, #1027) was used as control throughout. The dose-response curve for myocardin was done by incubating hCASMC with 1, 3, 10, 30, 100 and 300 MOI Ad-MYOCD-HA for 96h before harvesting. The effect of CCG-1423 (10 μM), which binds to RPEL motifs in MRTFs and inhibits their activation, was tested in serum starved cells. For knock-down of the serum response factor (SRF), a cocktail of GapmeRs (Exiqon, Design ID: 423124–1, 423124–2, 423124–3, 423124–4) against SRF was used at a final concentration 25 nM for each. hCASMC were seeded in 6 well-plates and after 24h, they were transfected with the GapmeR cocktail or 100 nM Negative control A (Exiqon) using Oligofectamine transfection reagent (Invitrogen). Transfection was performed in Opti-MEM Reduced-Serum Medium (LifeTechnologies). After 15h, the medium was exchanged for 231 medium supplemented with 5% SMGS and 50U/50μg/ml penicillin/streptomycin. After 72h in this medium, cells were starved for 24h (2% SMGS) and subsequently harvested at 96h.

For comparison of intact tissue and cultured cells from the same tissue we used mouse aorta. Mice (4–5 months old and of both sexes) were sacrificed with increasing CO_2_ and the abdomen and thorax were opened longitudinally. The intestines and the heart and lungs were removed *en bloc*. The aorta was next excised and immersed in cold Ca^2+^-free HEPES-buffered Krebs solution (in mM: NaCl 135.5, KCl 5.9, MgCl_2_ 1.2, glucose 11.6, HEPES 11.6, pH 7.4). Aortae were stripped of adhering fat and connective tissue under a dissection microscope and divided in two equal halves, one of which was opened longitudinally for removal of blood and immediately frozen in liquid N_2_. Cells were isolated from the other half by trypsin digestion as described (Ekman et al., 2013). Isolated cells were then cultured in DMEM/Ham’s F12 supplemented with antibiotics (penicillin and streptomycin) and 10% fetal calf serum (FCS) and harvested after 2–3 passages.

### RNA isolation and qRT-PCR

Cultured cells were washed twice with ice-cold phosphate buffered saline (PBS, Biochrom) and lysed in Qiazol (Qiagen). Total RNA was extracted using Qiagen miRNeasy mini kit (Qiagen #217004) in a QIAcube (Qiagen) according to manufacturer’s instructions. The concentration and quality of RNA was assessed using a Nanodrop spectrophotometer (Thermo Scientific). PCR reactions were performed using the Quantifast SYBR Green RT-PCR kit (Qiagen, #204156) and Quantitect (Qiagen) primer assays for: 18S, *PTRF*, *SDPR*, *PRKCDBP*, *MURC*, *CAV1*, *SRF*, *CAV2*, *CAV3*, *CNN1*, *EHD2* and *PACSIN2*. Primer sequences are proprietary information of Qiagen. Expression of mRNAs was measured using a real time thermal cycler (StepOnePlus, Applied Biosystems). In pilot experiments we examined C_T_ values for 18S, *GAPDH* and *BACT* after treatment with Ad-CMV-null and Ad-CMV-MYOCD, respectively. The C_T_ values for 18S (4.05±0.05 vs. 4.05±0.02) and *BACT* (30.3±0.7 vs. 29.7±0.3) were unchanged by MYOCD whereas the C_T_ value for *GAPDH* increased (17.1±0.1 vs. 15.8±0.07, P<0.01). We therefore used 18S for normalization throughout.

### Protein extraction and western blotting

Cells were washed two times with ice-cold PBS and lysed on ice in 50–60μl of 1X Laemmli sample buffer (60 mM Tris-Hcl, pH 6.8, 10% glycerol, 2% SDS). Protein determination was performed using the Biorad DC protein assay and 0.01% bromophenol and 5% β-mercaptoethanol were added. Western blotting was performed as described [48]. Briefly, 20 μg protein was loaded per lane on Bio-Rad TGX 4–15% or AnyKD Criterion gels. Following transfer to nitrocellulose, proteins were detected using the following antibodies: CAV1 (D46G3, Cell Signaling), CAV2 (610685, BD Transduction Laboratories), CAV3 (610421, BD Transduction Laboratories), Cavin-1/PTRF (ab48824, Abcam), Cavin-2/SDPR (ab113876, Abcam), Cavin-3/PRKCDBP (16250-1-AP, Proteintech), Tropomyosin/TPM1 (3910, Cell Signaling), SM22α/TAGLN (ab 14106, Abcam), HSP90 (610418, BD Transduction Laboratories) and GAPDH (MAB374, Millipore), all at recommended dilutions. Secondary HRP-conjugated anti mouse or anti rabbit antibodies (Cell Signaling) were then used for detection by enhanced chemiluminescence (Pierce West Femto). An Odyssey Fc Imager (LI-COR Biosciences) was used for image capture and Image Studio 3.1 was used for analysis.

### CAV1 promoter reporter assay

A LightSwitch promoter reporter for CAV1, containing 1077 nt of sequence ending at the transcription start site followed by a luciferase reporter, was purchased from Acive Motif (Nysdam, Belgium). Cells were cultured and transduced with Ad-MYOCD (as described above). 24h after transduction the CAV1 promoter reporter plasmid was transfected (Fugene 6, Promega) into hCASMC in 6-well plates. Cells were frozen (-80°C) 72h later. Cells were lysed in 100μl reporter lysis buffer (Promega) and luciferase activity was measured (Glomax, Promega) using the LightSwith Luciferase Assay System (Active Motif, Belgium).

### Immunohistochemistry of AngII-induced aneurysms

Mice were anaesthetized with 2% isoflurane and osmotic mini-pumps (model 2004, Alzet) loaded with AngII (1 mg/kg/min, Tocris) or saline were implanted subcutaneously (on the back) in male C57BL/6J mice under sterile conditions in adjoining laboratory spaces for microsurgery. An i.p injection of Temgesic (0.05mg/kg) was given at the time of pump implantation (8–12 a.m.) and the animals were checked continuously for any signs of pain or discomfort. Mini-pumps were used rather than daily injections to reduce stress. Two AngII-treated mice died from ruptured aneurysms after a week. Blood pressure was recorded (CODA, Kent Scientific) at zero and at three weeks (0w: 104±10 and 3w: 127±14 mm Hg, P<0.01), and aortae were harvested and cleaned following the last blood pressure recording. Three mice had developed aneurysms, two early and one with a diameter >2 mm, and these were cut with 1 mm rostrocaudal margins. Proximal and distal segments (5 mm each) were used as controls. Segments were immersed in 4% paraformaldehyde in phosphate-buffered saline (4h, room temperature) and processed for immunofluorescence staining using standard protocols [49]. Following permeabilization and antigen retrieval, cross sections (5 μm) were incubated with the following antibodies: CAV1 (D46G3, Cell Signaling, 1:100), PRKCDBP (Cavin-3, 16250-1-AP, Proteintech, 1:100) and CNN1 (Calponin, Abcam, 1:100). Targets were visualized using a donkey anti-rabbit antibody conjugated with Alexa-Fluor-555 (Invitrogen) at a dilution of 1:200 and nuclei were stained with DAPI (Invitrogen). Images were acquired using the Olympus CellSensDimension software and an Olympus DP72 microscope equipped with a digital camera. Staining for CAV1, PRKCDBP and CNN1 was done on consecutive serial sections.

### Electron microscopy

Human CASMC were seeded in 6-well plates with or without polycarbonate cell culture inserts (PIHP03050, Millipore). After treatment for 96h with 100 MOI of Ad-MYOCD-HA or Ad-CMV-null, cells were directly immersed in fixative followed by washing, post-fixation and processing as above. Sections were cut and examined in an electron microscope (JEOL 1230, Jeol, Tokyo, Japan) and digital micrographs were analyzed using Image J (NIH, Bethesda, MD, USA).

### Correlations using the GTEx database

In order to examine correlations between *MYOCD*/*MKL1*/*MKL2* and caveolae genes in different tissues, we used expression data available from the Genotype-Tissue Expression (GTEx) project (http://www.gtexportal.org/) [50]. Across-samples normalization for each tissue was performed using the trimmed mean of M-values (TMM) normalization method [51]. Correlations were calculated with the non-parametric Spearman method using R language for statistical computations [52]. Correlation matrices for each tissue were produced by R package "Hmisc".

### Other statistical analyses

All data are presented as means ±SEM. For most qRT-PCR presentations, individual data points are also depicted. GraphPad Prism Software 5 (GraphPad Software, San Diego California, USA) was used for statistical analyses. Significance was examined using student t-test or by one-way analyses of variance (ANOVA) followed by the Bonferroni post-hoc test for multiple comparisons. P <0.05 was considered significant.

## Results

### Caveolae genes are regulated by actin polymerization

**Fig 1:**
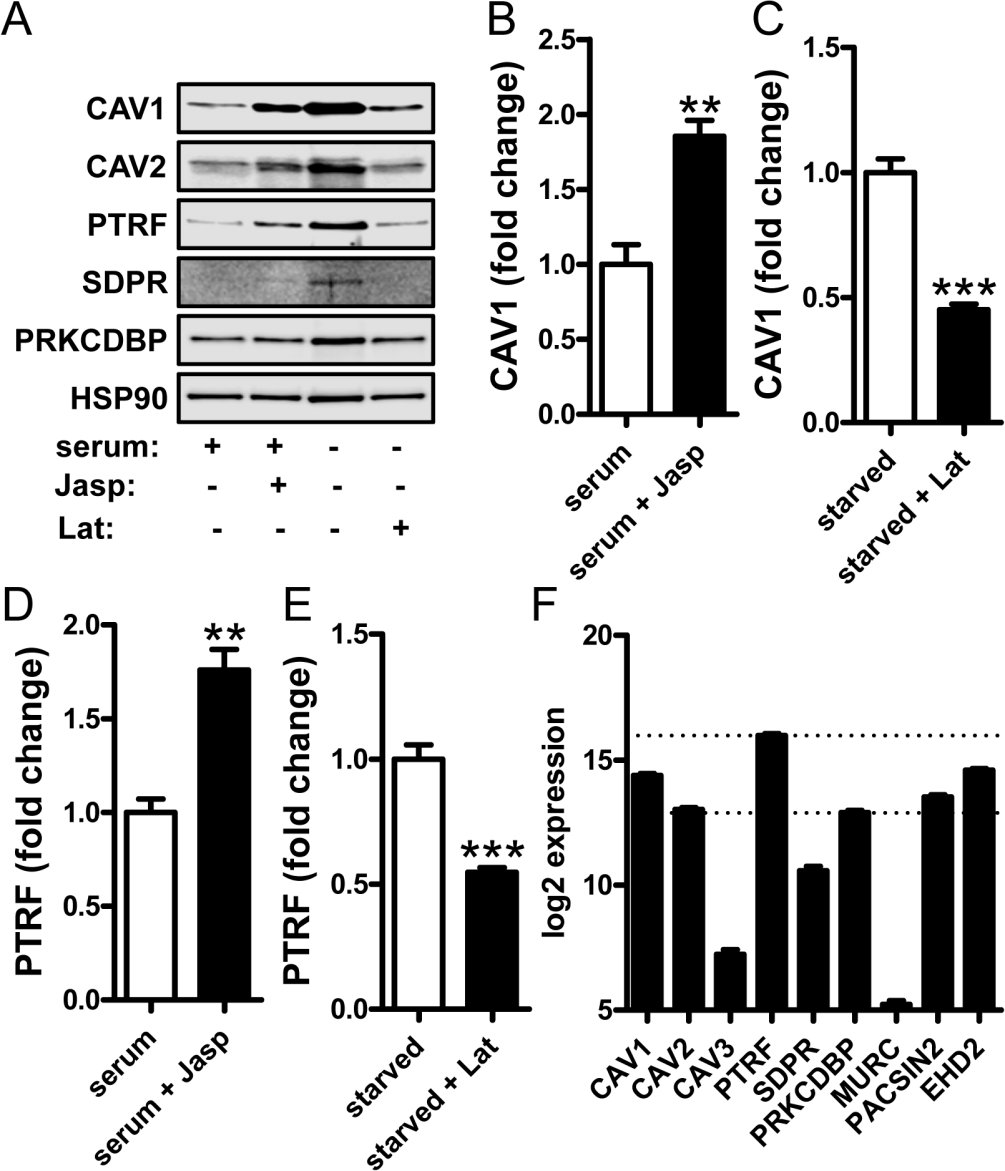
Caveolin and cavin protein expression is regulated by actin polymerization/ depolymerization. Human coronary artery smooth muscle cells (hCASMC) were treated with the actin stabilizing substance Jasplakinolide in the presence of 5% growth supplement “serum” or the actin de-polymerizing agent Latrunculin B in “starved” cells (2% growth supplement). Panel A shows representative western blots for caveolins (-1 and -2) and for cavins (-1, -2 and -3, official gene symbols: *PTRF*, *SDPR* and *PRKCDBP*). HSP90 was used as a loading control. Panels B-E show summarized western blot data for caveolin-1 and (B, C) and for cavin-1 (PTRF, D, E) in the presence of either Jasplakinolide or Latrunculin B, respectively. Panel F shows TMM-normalized mRNA read counts from 44 human coronary artery samples showing a comparatively low expression of *MURC* (cavin-4), *CAV3* and *SDPR*. Data are presented as means±S.E.M. **P<0.01, ***P<0.001. In panel F all differences are significant except *CAV1* vs. *EHD2*, *CAV2* vs. *PRKCDBP* and *CAV2* vs. *PACSIN2*.

The activity of two myocardin family members, MRTF-A (*MKL1*) and-B (*MKL2*), is controlled by changes in actin polymerization. This depends on their binding to globular (G)-actin in the cytosol which inhibits their nuclear translocation. We therefore first examined whether caveolae proteins are regulated by changes in actin polymerization. Primary human coronary artery smooth muscle cells (hCASMCs) were either treated with jasplakinolide, a drug that increases filamentous (F-) actin, or with latrunculin B which induces actin depolymerization. Due to poor stability of latrunculin B in serum, the latter treatment was done in serum-depleted media (2%), which in and of itself increased expression of caveolae proteins. CAV1 protein expression was increased by jasplakinolide and, in serum-depleted media, reduced by latrunculin B (Fig 1A, summarized data in B and C). Similar effects were seen for PTRF (cavin-1, Fig 1A and summarized data in D and E). PRKCDBP (cavin-3) was readily detectable (Fig 1A), but did not change significantly. We also blotted for CAV2 (Fig 1A), CAV3 (not shown), SDPR (cavin-2, Fig 1A) and MURC (cavin-4, not shown). With the exception of CAV2, which was regulated in the same manner as CAV1 (Fig 1A), these other caveolae proteins were difficult to detect (exemplified by blot for SDPR in Fig 1A). To address if this was due to low expression of CAV3, SDPR and MURC, TMM-normalized read counts from RNA sequencing of 44 coronary artery samples were plotted. As shown in Fig 1F, *CAV3*, *SDPR* and *MURC* were expressed at lower levels than other caveolae genes. They were therefore omitted from subsequent western blot analyses.

**Fig 2:**
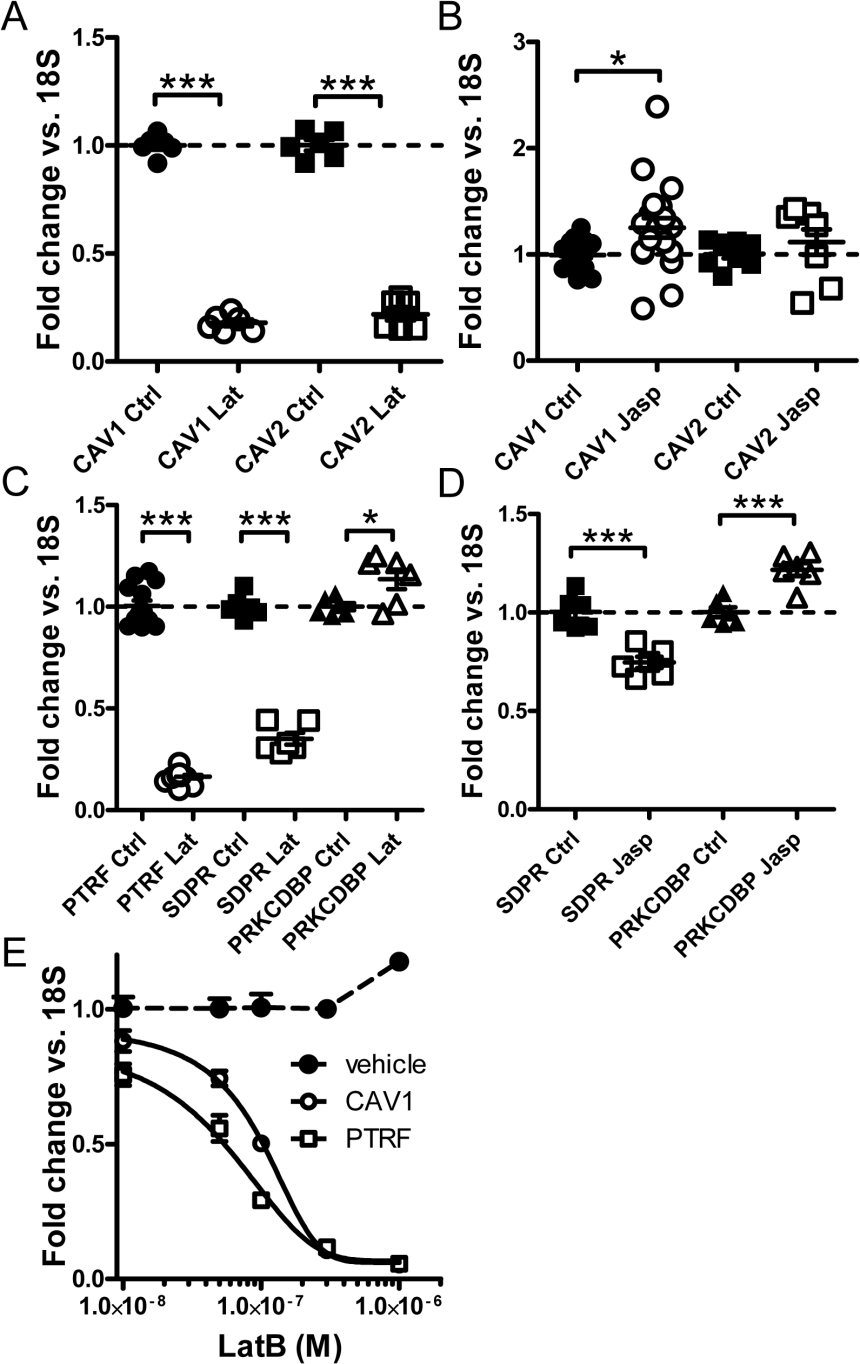
Actin depolymerization regulates caveolins and cavins at the mRNA level in human coronary artery smooth muscle cells. Cells were treated with either Latrunculin B (Lat; depolymerizes actin) or Jaspakinolide (Jasp; polymerizes actin) for 24h. qRT-PCR was performed to detect caveolin-1 (*CAV1*) and caveolin-2 (*CAV2*) mRNA levels, which were reduced by Lat (A) and only marginally affected by Jasp (B). Latrunculin B similarly reduced mRNA expression of cavin-1 and -2 (*PTRF* and *SDPR*) but slightly increased mRNA expression of cavin-3 (*PRKCDBP*, panel C). Treatment with Jasp did not affect cavin-1 (*PTRF*, not shown), modestly reduced the mRNA level of cavin-2 and slightly increased the mRNA expression of cavin-3 (D). 18S was used as housekeeping gene throughout. Panel E shows concentration-response curves for LatB and vehicle (DMSO). *CAV1* and *PTRF* were measured at each Lat concentration and compared to vehicle-treated (DMSO) control cells. Controls for *CAV1* and *PTRF* did not change with increasing DMSO (except at the highest concentration of DMSO) and were pooled for simplicity. Data are presented as means±S.E.M throughout. *P<0.05, **P<0.01, ***P<0.001.

The observed effects of drugs that affect actin polymerization could in principle depend on altered protein synthesis, altered protein degradation or both. There is ample evidence in the literature that caveolae proteins are regulated at the level of protein degradation. We found however that depolymerization of actin with latrunculin B reduced the mRNA levels for *CAV1* and *CAV2* (5-fold, Fig 2A) as determined by qRT-PCR. Polymerization of actin with jasplakinolide modestly increased *CAV1* whereas the effect on *CAV2* failed to reach significance (Fig 2B). Latrunculin B also reduced expression of *PTRF* and of *SDPR* (5-fold, Fig 2C). *PRKCDBP* (cavin-3) expression was instead slightly increased. Actin polymerization with jasplakinolide failed to induce *PTRF* (not shown) and slightly reduced expression of *SDPR* (Fig 2D). Modest induction of *PRKCDBP* was however seen (Fig 2D). The discrepant effect of jasplakonolide on PTRF protein and mRNA was addressed in an experiment where protein synthesis was inhibited by cycloheximide (not shown). While PTRF tended to be stabilized by jasplakinolide at an early time (P = 0.07 at 42h), the drug combination was cytotoxic upon longer incubation, precluding in depth characterization of any protein stabilizing effect. Concentration-response curves indicated slightly higher sensitivity of *PTRF* than of *CAV1* to latrunculin B (Fig 2E). Time-course studies disclosed maximal *PTRF* repression at 12h whereas maximal *CAV1* repression was seen at 24h (not shown); repression then subsided with time but was maintained for at least 48h in both cases. We therefore conclude that the mRNA expression of caveolins and cavins is regulated by latrunculin B-induced actin depolymerization.

### Caveolae genes are regulated by myocardin family coactivators

**Fig 3:**
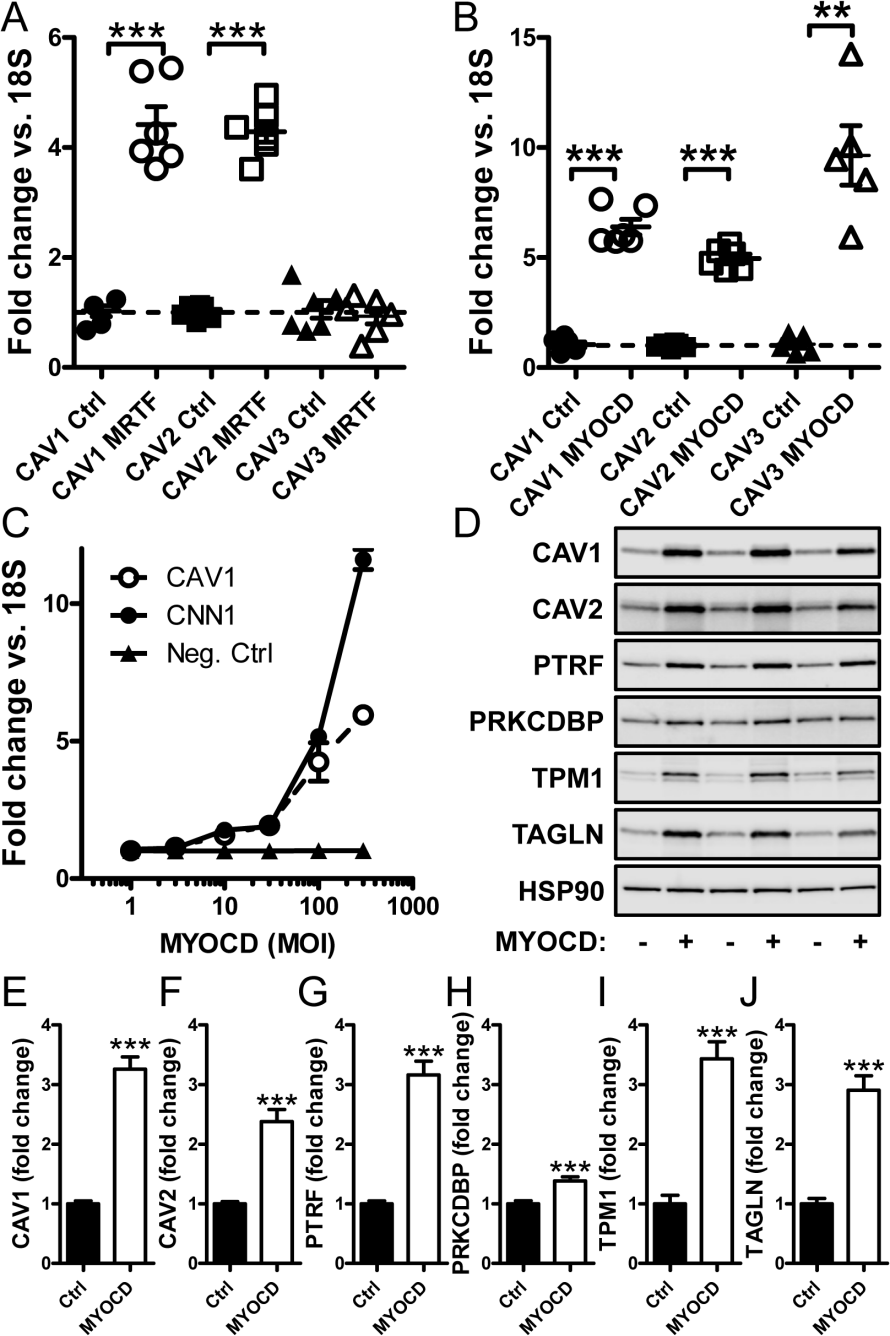
Induction of caveolins by adenoviral transduction of MRTF-A and myocardin. (A) Adenoviral transduction of human coronary artery smooth muscle cells with MRTF-A (*MKL1*, 20 MOI) increased expression of caveolin-1 and caveolin-2, but not caveolin-3 mRNA compared to empty virus. (B) Transduction of myocardin (MYOCD, 100 MOI) increased mRNA expression of all three caveolins (-1 to -3). (C) A dose-response curve with increasing concentrations of myocardin (1 to 300 MOI) demonstrated a similar threshold of sensitivity of caveolin-1 mRNA to that of the prototypical myocardin target calponin (*CNN1*). (D) Western blotting of MYOCD-transduced cells demonstrated increases of caveolins and cavins at the protein level. The magnitude of induction was similar to that of the well-established myocardin targets tropomyosin (*TPM1*) and SM22α (*TAGLN*). Summarized data from the western blots is shown in panels E through J (n = 8). 18S was used as a house-keeping gene for the qRT-PCR and HSP90 as loading control for western blotting. Data are presented as means±S.E.M. **P<0.01, ***P<0.001.

The high sensitivity of caveolin and cavin synthesis to latrunculin B could reflect involvement of myocardin family coactivators [29, 47]. We therefore next tested if caveolins and cavins are transcriptional targets of these coactivators. MRTF-A (*MKL1*) and myocardin (*MYOCD*) were overexpressed using adenoviruses. Compared to empty vector, transduction of MRTF-A (20 MOI) in the presence of serum caused 4-fold induction of *CAV1* and *CAV2* while *CAV3* was unchanged (Fig 3A). Transduction of myocardin (100 MOI) increased the mRNA levels for all caveolins with close to 10-fold induction of *CAV3* (Fig 3B).

Next, the induction of *CAV1* by increasing doses of MYOCD was compared to that of another gene known to be induced by MYOCD: calponin (*CNN1*). We found similar thresholds of induction for *CAV1* and *CNN1*, but *CNN1* induction was somewhat greater than that of *CAV1* at the highest MOI (Fig 3C). We further examined if adenoviral transduction of MYOCD affected the levels of caveolae proteins. Transduction of MYOCD increased the levels of caveolins (1 and 2) and of cavin-1 at least 3-fold (Fig 3D), which was similar to the prototypical MYOCD targets tropomyosin (TPM1) and SM22α (TAGLN) (Fig 3E–3J). Cavin-3 (PRKCDBP) was modestly but significantly increased at the protein level following myocardin transduction (Fig 3D and 3H).

**Fig 4:**
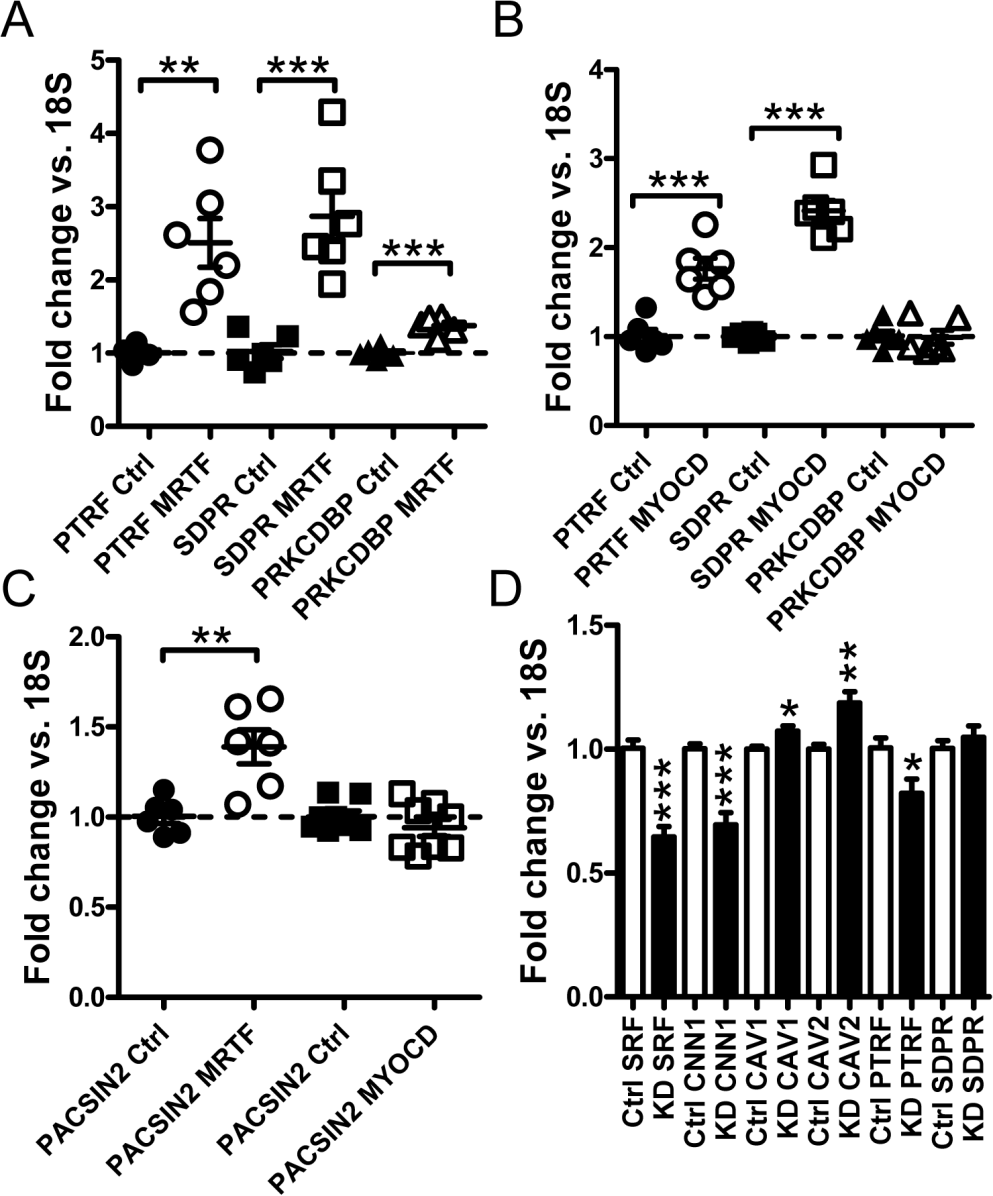
Myocardin and MRTF-A transduction increases cavin mRNA levels. (A) Adenoviral transduction of hCASMC with MRTF-A (*MKL1*) elevates the mRNA expression of cavin-1, -2 and -3 (*PTRF*, *SDPR* and *PRKCDBP* respectively). (B) Adenoviral transduction with myocardin resulted in upregulation of cavin-1 and -2 but not of cavin-3 mRNA. (C) MRTF but not myocardin induced *PACSIN2* mRNA expression. (D) mRNA expression for *SRF*, *CNN1*, *CAV1*, *CAV2*, *PTRF* and *SDPR* in cells transfected with negative control GAPmers (white bars) and with GAPmers targeting *SRF* (black bars). Data are presented as means±SEM. *P<0.05, **P<0.01, ***P<0.001.

The effect of MRTF-A and MYOCD transduction on cavin mRNA levels was next examined. Both MRTF-A (Fig 4A) and MYOCD (Fig 4B) induced *PTRF* and *SDPR*. MRTF-A had a small but significant effect on *PRKCDBP*, but MYOCD was without effect on this gene. We also tested whether *MURC* (cavin-4), which is enriched in striated muscles, was induced. If anything, MYOCD caused slight repression of *MURC* (data not shown). *PACSIN2* which regulates membrane curvature in caveolae was induced by MRTF-A, but this gene was unaffected by myocardin (Fig 4C).

Our next step was to knock down serum response factor (SRF), which mediates many of the effects of the myocardin family coactivators. We used a mixture of SRF-targeted Gapmers to achieve 40% knockdown of *SRF* mRNA in hCASMCs (Fig 4D). As expected, reduction of *SRF* was associated with a reduction of the prototypical MYOCD/SRF target *CNN1*. Knockdown of SRF modestly reduced cavin-1 (*PTRF*) expression but expression of *CAV1* and *CAV2* (Fig 4D) was increased. *SDPR* expression was not affected.

**Fig 5:**
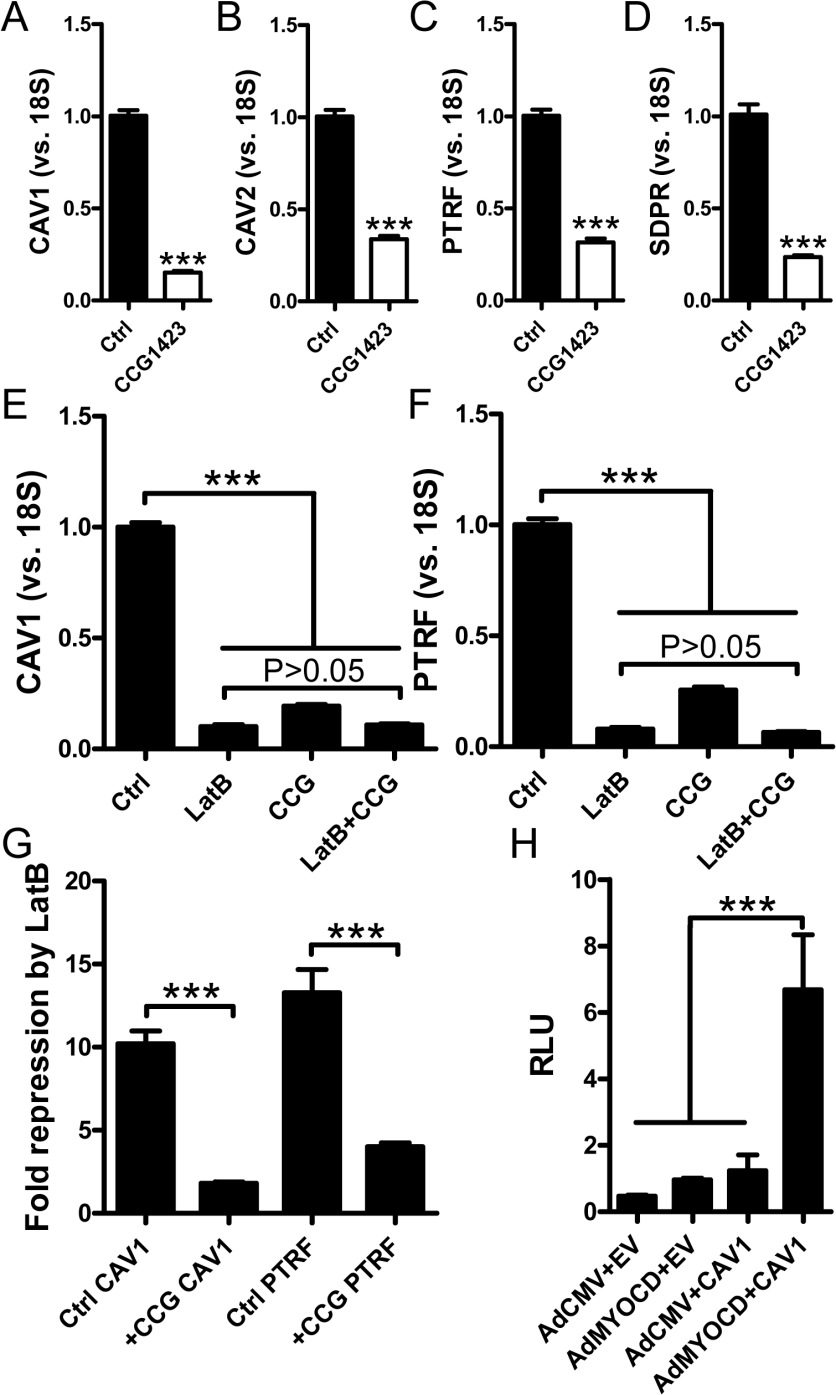
CCG-1423 mitigates the effect of actin depolymerization and MYOCD acts via the proximal *CAV1* promoter. Panels A-D show effects of the MRTF (MKL) inhibitor CCG-1423 on caveolin and cavin mRNA expression in serum-depleted human coronary artery smooth muscle cells. 18S was used as a house-keeping gene for the qRT-PCR. Panels E (*CAV1*) and F (*PTRF*) shows an experiment where CCG-1423, latrunculin B and their combination were run in parallel with vehicle-treated controls. (G) Data in panels E and F was used to calculate fold repression by latrunculin B in the absence and presence of CCG-1423. Panel H shows a *CAV1* promoter reporter assay. Three negative controls were included: cells transfected with empty vector (EV) followed by treatment with Ad-CMV-Null or Ad-CMV-MYOCD, as well as cells transfected with the CAV1 reporter plasmid (CAV1) followed by treatment with Ad-CMV-Null. Relative luciferase activity (RLU: relative luciferase units) was increased ≈7-fold by MYOCD in cells containing the reporter.

To further support involvement of myocardin-related coactivators in basal expression of caveolins and cavins, we used CCG-1423 (10 μM). This substance binds to RPEL motifs in MRTFs and inhibits their nuclear translocation thus providing for a pharmacological loss of function approach. In serum-depleted media, CCG-1423 inhibited expression of *CAV1*, *CAV2*, *PTRF* and *SDPR* 3-5-fold (Fig 5A–5D). We also examined if the effects of CCG-1423 and latrunculin B were additive. These experiments showed that latrunculin B and CCG-1423 were almost equipotent (Fig 5E and 5F) and that repression of *CAV1* and *PTRF* by latrunculin B was almost absent in the presence of CCG-1423 (Fig 5G). This indicates that myocardin family coactivators mediate the effect of actin depolymerization on caveolin/cavin expression.

Using a *CAV1* promoter reporter we next addressed if *CAV1* regulation by MYOCD is mediated by the proximal promoter (1077 nt of 5’ sequence). MYOCD transduction had no effect in cells transfected with empty vector but increased luciferase activity 7-fold in cells transfected with reporter (Fig 5H). This argues MYOCD regulation of *CAV1* is direct, and that full regulation is retained in the proximal promoter.

### The density of caveolae is increased following transduction with MYOCD

**Fig 6:**
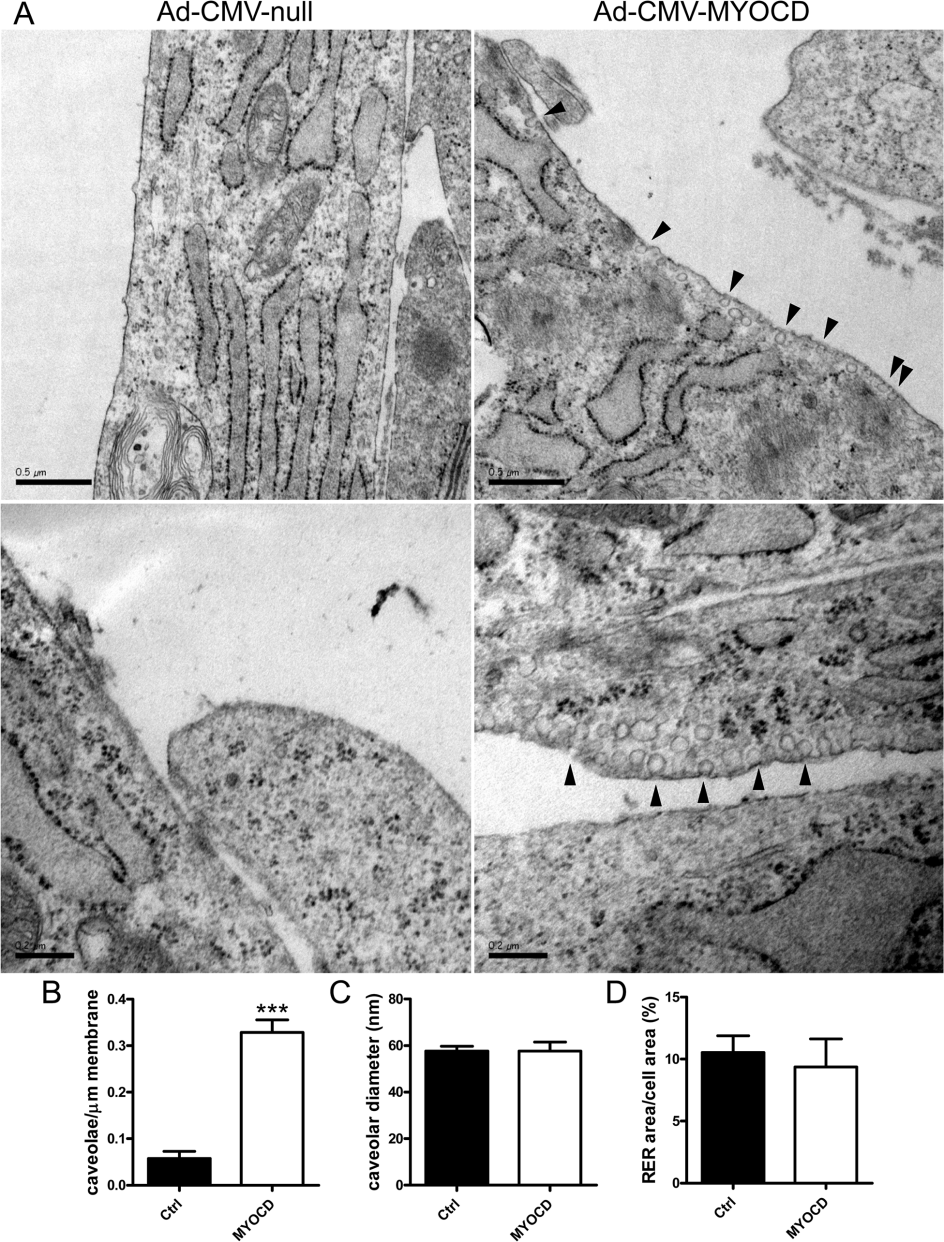
Myocardin drives formation of caveolae. Coronary smooth muscle cells, cultured on polycarbonate membranes, were transduced with Ad-CMV-MYOCD or Ad-CMV-null for 96h and prepared for electron microscopy. Left micrographs in panel A show control cells (Ad-CMV-null) where caveolae were rare. Rough endoplasmic reticulum was very prominent in these cultured cells. Right micrographs show caveolae in cells transduced with MYOCD (black arrowheads). Panel B shows quantification of the number of caveolae per μm membrane in cells from three independent transductions. The total membrane lengths examined for Ad-CMV-null and Ad-CMV-MYOCD were 501 and 340 μm, respectively. Panel C shows the diameters of caveolae in control and MYOCD transduced cells. Panel D shows area of rough endoplasmic reticulum compared to total cell area. Scale bars in the upper micrographs of panel A represent 500 nm whereas those in the lower micrographs represent 200 nm.

Our findings so far established that myocardin-related coactivators control the expression of genes from at least three families of importance for the biogenesis of caveolae. We therefore directly examined if myocardin drives formation of caveolae. hCASMCs were transduced with adenovirus encoding myocardin or empty vector. They were then processed for electron microscopy and the density of caveolae was determined. Overexpression of myocardin led to a higher density of membrane caveolae compared with control (Fig 6A). Summarized data established a 5-fold increase of the density of caveolae after transduction of myocardin (bar graph in Fig 6B). The diameters of caveolae were similar (Fig 6C) and the relative area of the endoplasmic reticulum was unchanged (Fig 6D).

### Expression of caveolins and cavins is reduced in cultured cells and after arterial injury

**Fig 7:**
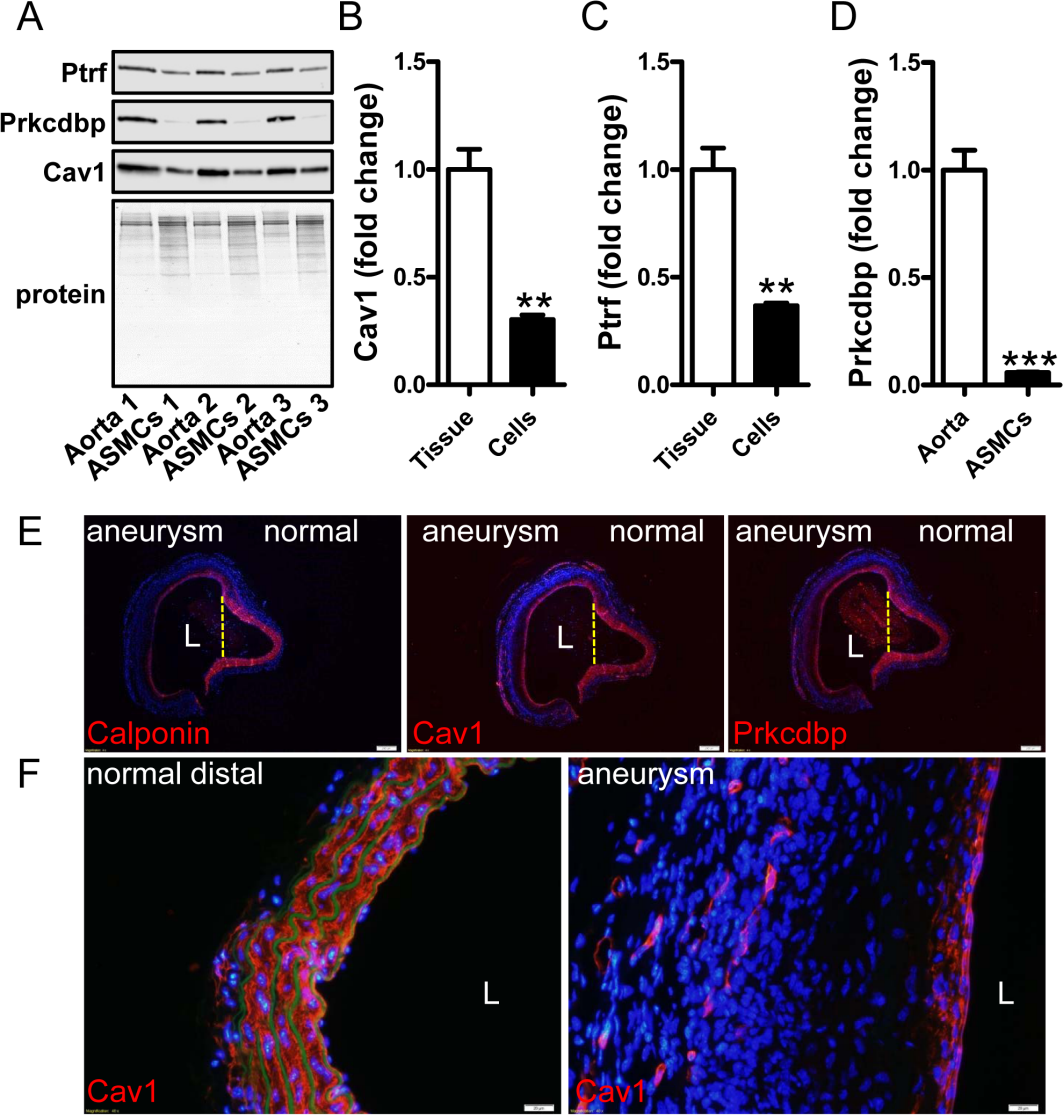
Caveolin-1 and cavin-1 conform to patterns of regulation established for classical smooth muscle differentiation markers. Aortae from three different control mice were isolated. One piece was immediately frozen and the other was used for isolating smooth muscle cells which were subsequently cultured *in vitro*. (A) Western blots show higher expression of cavin-1 (Ptrf) and -3 (Prkcdbp) and caveolin-1 (Cav1) in whole aortae than in cultured cells from the same vessels. Panels B-D show summarized western blot data. Data are presented as means±SEM.**P<0.01, ***P<0.001. Panel E shows staining (in red) for calponin (left), caveolin-1 (middle) and cavin-3 (Prkcdbp, right) in consecutive sections from a saccular aortic aneurysm induced by AngII. This aneurysm had a diameter exceeding 2 mm and the lumen is indicated by an L. The aneurysm bulges to the left whereas the right side is normal. The partitions between normal and diseased areas are indicated by dotted yellow lines. A progressive reduction, from the normal to the dilated side, of medial staining for calponin, caveolin-1 and cavin-3 is evident, leaving only a thin red rim just below the endothelium at the aneurysmal site. This is shown at higher magnification in panel F, giving views of caveolin-1 staining in distal control aorta (left) and in the aneurysm (right). Caveolin-1-positive capillary structures are present in the adventitia of the aneurysm whereas medial staining is drastically reduced. Nuclei were stained with DAPI throughout. In panel F, autofluorescence (green) was used to visualize elastic lamellae showing loss of elastic fibers in the aneurysm to the right. Images are representative of three aneurysms and three control aortae. Scale bars (white) in E and F represent 200 and 20 μm, respectively.

Our findings argued that several caveolae genes share transcriptional regulatory mechanisms with classical smooth muscle differentiation marker genes. Smooth muscle markers, including the MYOCD coactivator, are known to decrease when cells are isolated and cultured *in vitro* as well as in various injury models *in vivo* [53]. We therefore next addressed if selected caveolae proteins follow this pattern of regulation. First we examined expression of caveolin-1, cavin-1 and cavin-3 in intact mouse aortae and in cultured cells from the same specimens. This demonstrated reductions of these proteins compared to total protein remaining on the gels after transfer (Fig 7A–7D). We also examined caveolin-1 and cavin-3 staining in angiotensin II-induced aortic aneurysms in mice (the cavin-1 antibody was not ideal for staining of mouse tissues). Blood pressure was significantly elevated in angiotensin II treated mice (104±9.6 (day 0) vs. 127±14mmHg (day 21)) and weight gain was similar in both groups (3.7±0.5 (saline) vs 2.6 ±0.7g (Ang II)). Similar to the myocardin target calponin (red in left panel of Fig 7E), reduced staining for caveolin-1 and cavin-3 was seen in all three aneurysms compared to adjacent normal media (red in the two panels to the right in Fig 7E). This was also evident when aneurysms were compared with distal normal aorta at higher magnification (Fig 7F, right vs. left). Caveolins and cavins therefore adhere to patterns of expression seen for classical smooth muscle differentiation markers.

### mRNA expression of myocardin family members correlate with expression of caveolae genes in human tissues

**Fig 8:**
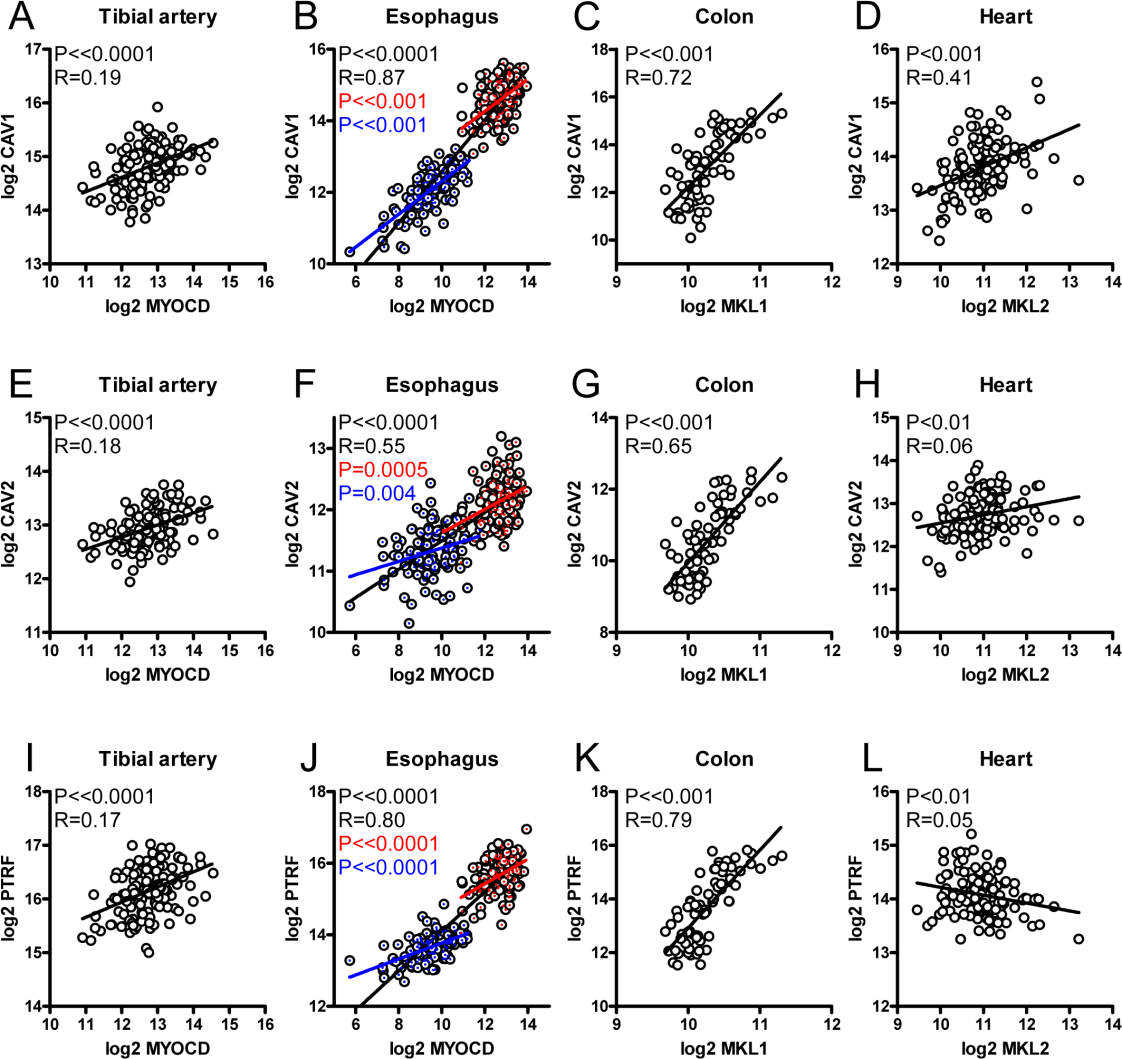
Expression of myocardin family genes correlate with expression of caveolae genes in humans. mRNA expression data was retrieved from the GTExPortal. TMM-normalized data was used to correlate expression of myocardin family genes with *CAV1*, *CAV2* and *PTRF* in the tibial artery (panels A, E, I, n = 137), in esophagus (B, F, J, n = 227), in colon (C, G, K, n = 74) and in heart (D, H, L, n = 134). P-values and Spearmann Rho coefficients (R) are given in the respective panels. In all datasets where more than one tissue were represented, such as the esophagus (muscularis: red symbols; mucosa: blue symbols), we always observed significant correlations both within (red and blue P-values) and across (black P-values) tissues.

Myocardin-related coactivators may contribute to *CAV1* variation in human tissues. To test this, mRNA expression data for caveolins, cavins and myocardin-related transcription factors was retrieved from the GTEx Portal. Myocardin (*MYOCD*) correlated significantly with *CAV1* in a majority of human tissues as exemplified by tibial artery (Fig 8A) and esophagus (Fig 8B). In esophagus, correlations were seen both in the mucosa (blue in Fig 8B, 8F and 8J), in the muscularis (red), and in both tissues combined (black), arguing that MYOCD explains variation not only within but also between tissues. *MKL1* (MRTF-A) also correlated with *CAV1* in many tissues such as in colon (Fig 8C). Instances of negative correlations between *MKL1* and *CAV1* were occasionally observed. This is perhaps to be expected from competition between myocardin and MRTF-A. In heart (Fig 8D) and adipose tissue (not shown) only *MKL2* correlated with *CAV1*. Similar correlations were seen for *MYOCD* vs. *CAV2* and *MYOCD* vs. *PTRF* (Fig 8, panels E-L), but rare exceptions were seen (Fig 8L). A summary of the correlations observed in these tissues is provided in Table 1. Together, these observations argue that myocardin family coactivators represent a quantitatively important transcriptional control mechanism for caveolae genes in human tissues. Supporting the notion that these genes are coregulated, we also found correlations between *CAV1* and *CAV2*, between *CAV1* and *PTRF* and between *CAV1* and *SDPR* in these tissues (S2 Fig, panel A). *CAV1* always correlated better with *CAV2* than with *SDPR* (c.f. S2 Fig, tibial artery or heart).

**Table 1:** Correlations of MYOCD, MKL1 and MKL2 versus CAV1 in different human tissues.

	Tibial artery			Esophagus			Colon			Heart		
	*MYOCD*	*MKL1*	*MKL2*	*MYOCD*	*MKL1*	*MKL2*	*MYOCD*	*MKL1*	*MKL2*	*MYOCD*	*MKL1*	*MKL2*
Significant positive	X	X	X	X	X	X	X	X	X			X
No correlation										X	X	
Significant negative												

### Role of chromatin remodeling in regulation of caveolae genes

**Fig 9:**
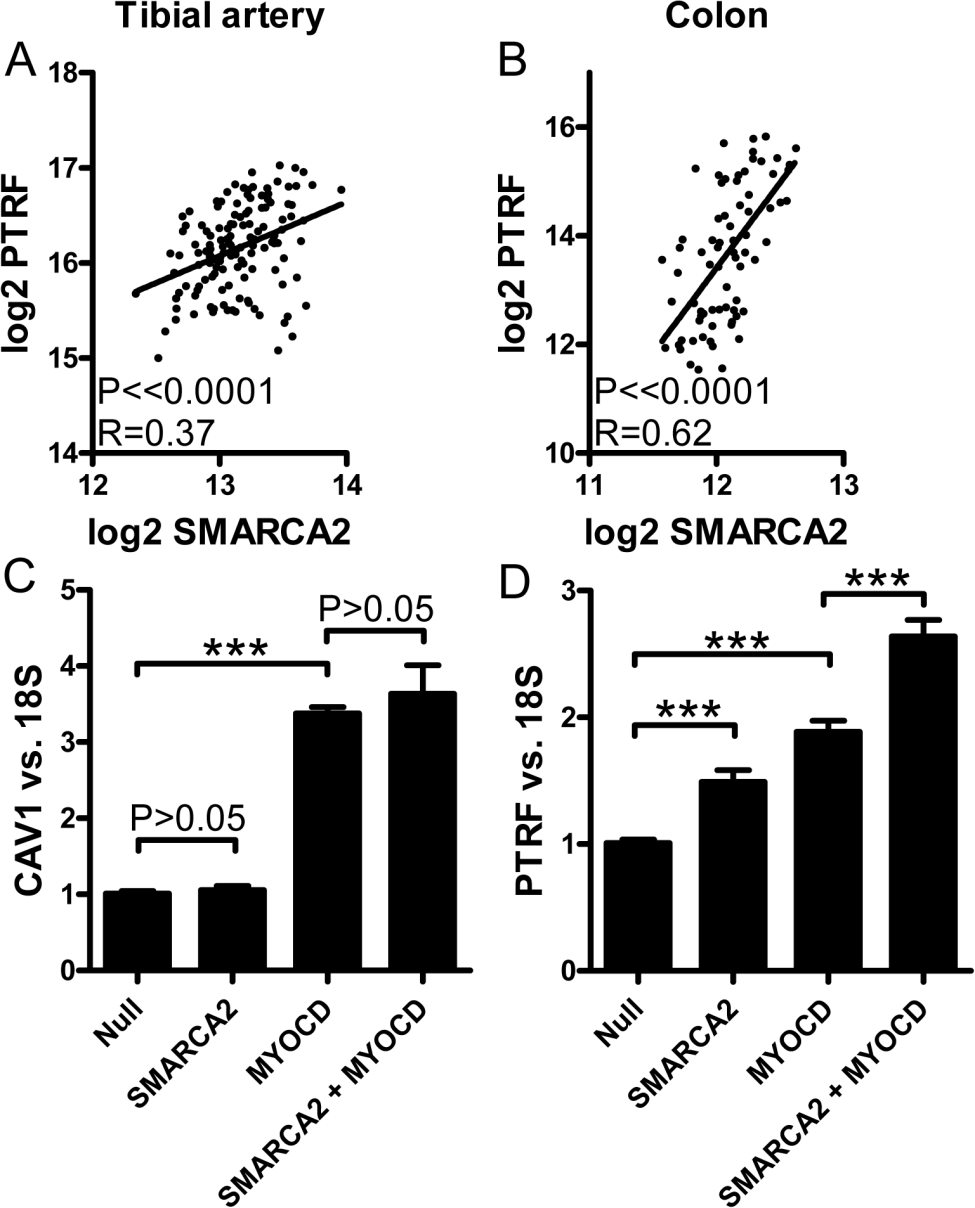
Chromatin remodeling plays role for the effects of myocardin family proteins on caveolin and cavin expression. Panels A and B show correlations between mRNAs for one component of the SWI/SNF chromatin remodeling complex (*SMARCA2*) and *PTRF* in tibial artery and colon, respectively. Panels C and D shows effect of adenoviral overexpression of SMARCA2 (hBrm) on *CAV1* and *PTRF*, respectively, in human coronary artery smooth muscle cells.

Prior work has demonstrated that myocardin family proteins recruit a SWI/SNF chromatin remodeling complex to SRF target genes and that this is important for their regulation. In keeping with this possibility we found that *SMARCA2* (Brm) correlated with *PTRF* in tibial artery and colon in the GTEx database (Fig 9A and 9B). To support a cause and effect relationship experimentally we overexpressed *SMARCA2* (Brm), both alone and together with *MYOCD*. No effect was seen on *CAV1* (Fig 9C). For *PTRF*, on the other hand, we found significant transcriptional activation and an additive effect with *MYOCD* (Fig 9D).

## Discussion

Genetic on-off switches for generation of caveolae need to account for the unique tissue distribution of caveolae and for the coordination of caveolin and cavin synthesis. Members of the myocardin protein family potentially meet both of these prerequisites. First, myocardin family proteins play roles in striated and smooth muscle, in endothelial cells and in adipocytes [35–37, 53, 54], all of which are cell types characterized by high densities of caveolae. Second, we find that caveolin and cavin synthesis is coordinately regulated by two myocardin family coactivators. We therefore conclude that myocardin family proteins likely meet critical requirements for a major genetic control mechanism of caveolae biogenesis.

The effects of myocardin family coactivators on caveolae genes is likely to be direct as the sensitivity and magnitude of protein and mRNA induction compared well with that of prototypical target genes. For CAV1 we also demonstrate that the full effect appears to be retained in the proximal promoter. In agreement with a recent microarray study demonstrating reduced expression of *PTRF* after *SRF* knock down [55], we found that *PTRF* was sensitive to *SRF* silencing (the closest CArG box in cis that binds SRF at the *PTRF* locus is depicted in S2B Fig), supporting a direct effect also in this case. SRF-dependence could not be demonstrated for *CAV1*, *CAV2* or *SDPR*, arguing that they are regulated by myocardin’s SAP domain independently of SRF. This conclusion is in keeping with recent analyses of the proximal *CAV1* promoter, failing to identify any CArG boxes [56]. The SAP domain has been demonstrated to mediate induction of tenascin-C independently of SRF [28], and is moreover known to play a role for regulation of atrial natriuretic factor [57], but the DNA motif to which it binds is poorly defined. Recent work by us has demonstrated that the α8 integrin gene (Itga8) is regulated by myocardin independently of SRF [58]. Three of the caveolae genes (*CAV1*, *CAV2* and *SDPR*) thus seem to belong to a growing group of myocardin family targets that are regulated by myocardin independently of SRF. A dual requirement of SRF and myocardin for regulation of *PTRF*, but not for regulation of *CAV1*, suggests a mechanism for uncoupling of *PTRF* synthesis from *CAV1* synthesis, such as seen in prostate cancer cells [59].

Our demonstration that MYOCD/MKL1 regulates the mRNA levels of several caveolins and cavins raised the possibility that these coactivators may singlehandedly control the genesis of caveolae. We directly addressed this using electron microscopy and were able to demonstrate an increase of the density of caveolae after myocardin transduction with no change of caveolar geometry. The 5-fold increase of caveolar density compared well with the effect of myocardin on *CAV1* mRNA and protein (4-6-fold), but appeared greater than the effect on *PTRF* mRNA (<2-fold). A possible explanation is that *CAV1* is the limiting factor and that surplus PTRF, not stabilized by the caveolar scaffold, is broken down. In support of this notion, we found that *PTRF* mRNA was higher than *CAV1* mRNA in human coronary arteries. In aggregate, our findings support an important role of myocardin for biogenesis of caveolae, but it should be noted that the density of caveolae in cultured smooth muscle cells is almost an order of magnitude lower than in intact arteries [9]. More protracted time-courses as well as *in vivo* knockout approaches are required to fully appreciate the magnitude of regulation of caveolar density by myocardin-related coactivators.

Prior studies have demonstrated several transcriptional control mechanisms for both caveolins and cavins, but no single described mechanism readily explains the tissue distribution of caveolae. In this regard we believe that the myocardin family proteins have considerable potential. In an attempt to support the hypothesis that variations in *MYOCD* underlie variations in *CAV1*, we used publicly available mRNA expression data and were able to demonstrate remarkable correlations in most human tissues. It was clear from these analyses, however, that *MYOCD*/*MKL1* cannot explain variation of *CAV1* in certain tissues. Notable examples are heart and subcutaneous adipose tissue where instead *MKL2* correlated significantly with *CAV1*. Work to examine if other myocardin-like transcription factors, including both *MKL2* and *MASTR*, may independently drive formation of caveolae is therefore highly warranted.

Our demonstration that caveolin-1 and cavin-1 are regulated by myocardin allows for formal categorization of these proteins as smooth muscle differentiation markers. Proteins in this category include myosin heavy chain, tropomyosin, calponin and SM22α. All of these proteins share the property that they are downregulated when smooth muscle cells undergo phenotypic modulation from a differentiated contractile to a proliferative or synthetic phenotype. This switch is known to reflect reduced myocardin expression [53] as well as increased competition from ternary complex factors for a common binding site on SRF [27]. SMC phenotypic modulation occurs in vascular diseases such as neointima formation and atherosclerosis and when SMC are isolated and cultured *in vitro* [53, 60]. Caveolin-1 has previously been shown to follow this pattern of regulation, being reduced in arterial lesions such as in atherosclerotic plaques [61] and after wire injury *in vivo* [49]. Work has also shown that SMC cultured *in vitro* lose caveolae [62]. The latter study however failed to demonstrate an accompanying reduction of caveolin expression by western blotting. This issue was therefore readdressed herein. In our hands, using mouse aorta, loss of caveolin-1 and cavin-1 expression was evident between intact tissue and cultured SMC from the same arteries. We also demonstrate that caveolin-1 expression is reduced in yet another form of arterial injury, namely aortic aneurysms. There is little doubt, therefore, that caveolins may be classified as *bona fide* SMC differentiation markers. In fact, myocardin family proteins are known to recruit the SWI/SNF chromatin remodeling complex to SRF-dependent smooth muscle marker genes [63] and we provide experimental support that this also applies for *PTRF*.

MRTF-A (*MKL1*) and MRTF-B (*MKL2*) communicate changes in the state of actin polymerization to the nucleus. This occurs by binding of these proteins to globular actin via RPEL-motifs. Even though myocardin is located in the cell nucleus, sensitivity to changes in actin polymerization may be conferred on this protein by hetero-dimerization with either MRTF-A or MRTF-B [29]. Here we found that actin polymerization and depolymerization resulted in reciprocal changes in caveolin and cavin protein levels. These proteins are therefore regulated via a biologically relevant control mechanism for myocardin family proteins. Actin depolymerization was considerably more effective at the mRNA level in our experiments. It is well established that certain MRTF targets only respond to actin depolymerization [64], but we suspect that the small effect of jasplakinolide at the mRNA level may be cell-type dependent and reflect basal expression of myocardin family proteins. An interesting and largely overlooked study demonstrated that Rho activation, which leads to actin polymerization, led to formation of caveolae in MC3T3-E1 cells [65], but the mechanism involved was not described. We are presently uncertain how to reconcile the increase of CAV2 and PTRF proteins after jasplakinolide with the lack of consistent effects of jasplakinolide on the mRNA level. Possibilities include stabilization of the caveolar scaffold via CAV1/PRKCDBP induction, or that a dynamic actin cytoskeleton is required for internalization and degradation of caveolae. In this regard it is interesting to note that EHD2 was recently demonstrated to link caveolae to the actin cytoskeleton [14]. We attempted to support a PTRF stabilizing effect of jasplakinolide in an experiment with the protein synthesis inhibitor cycloheximide, but the drug combination was cytotoxic ruling out any firm conclusions in this regard.

Our present results support the notion that the myocardin family of transcriptional coactivators plays a key role for genesis of caveolae, but for some caveolae genes the major regulatory influences likely remain to be identified. Cavin-3 (*PRKCDBP*), for example, was only modestly induced by MRTF-A (*MKL1*) and was unaffected by MYOCD. This is somewhat counterintuitive because cavin-3 has the most artery-specific expression pattern of all caveolins and cavins (c.f. http://www.gtexportal.org). Additional important control mechanisms also likely exist for cavin-2 (*SDPR*) which is expressed at a rather low level in arterial smooth muscle, being difficult to detect. Mechanisms that govern a high expression of SDPR in adipocytes (and some smooth muscles) therefore remain to be identified. It is formally possible that this is a reflection of different transactivation potential of myocardin family members at different gene loci, such as seen here for MRTF-A and MYOCD at the *CAV3* locus, but completely distinct mechanisms and combinatorial effects may also be involved.

This initial study raises a number of questions that need to be addressed in future work. For instance, work with domain deletion mutants is required to prove involvement of the SAP domain and genetic loss of function approaches are required to estimate the contribution of myocardin family members to formation of caveolae *in vivo*. If MRTF-B (MKL2) influences biogenesis of caveolae in adipose tissue and whether myocardin has an impact on the membrane lipid profile are other interesting questions. If MASTR, which lacks an SRF-binding domain but retains the SAP domain, is involved in biogenesis of caveolae in skeletal muscle is moreover of interest. These questions are being addressed in studies based on our present demonstration that caveolins and cavins are regulated at the transcriptional level by two myocardin family coactivators: myocardin and MRTF-A. *MYOCD* expression was moreover found to correlate with *CAV1* expression in several human tissues arguing that this transcriptional control mechanism is quantitatively important.

## Supporting Information

S1 Fig The ARRIVE guidelines checklist for reporting on animal *in vivo* experiments. (PDF)

S2 Fig Co-regulation of caveolins and cavins in smooth muscle and heart. (TIF)
