# Supplementary figures and images for: Myocardin Family Members Drive Formation of Caveolae
Source: PLoS One. 2015 Aug 5;10(8):e0133931. doi: 10.1371/journal.pone.0133931 (PMC4526231; doi:10.1371/journal.pone.0133931)

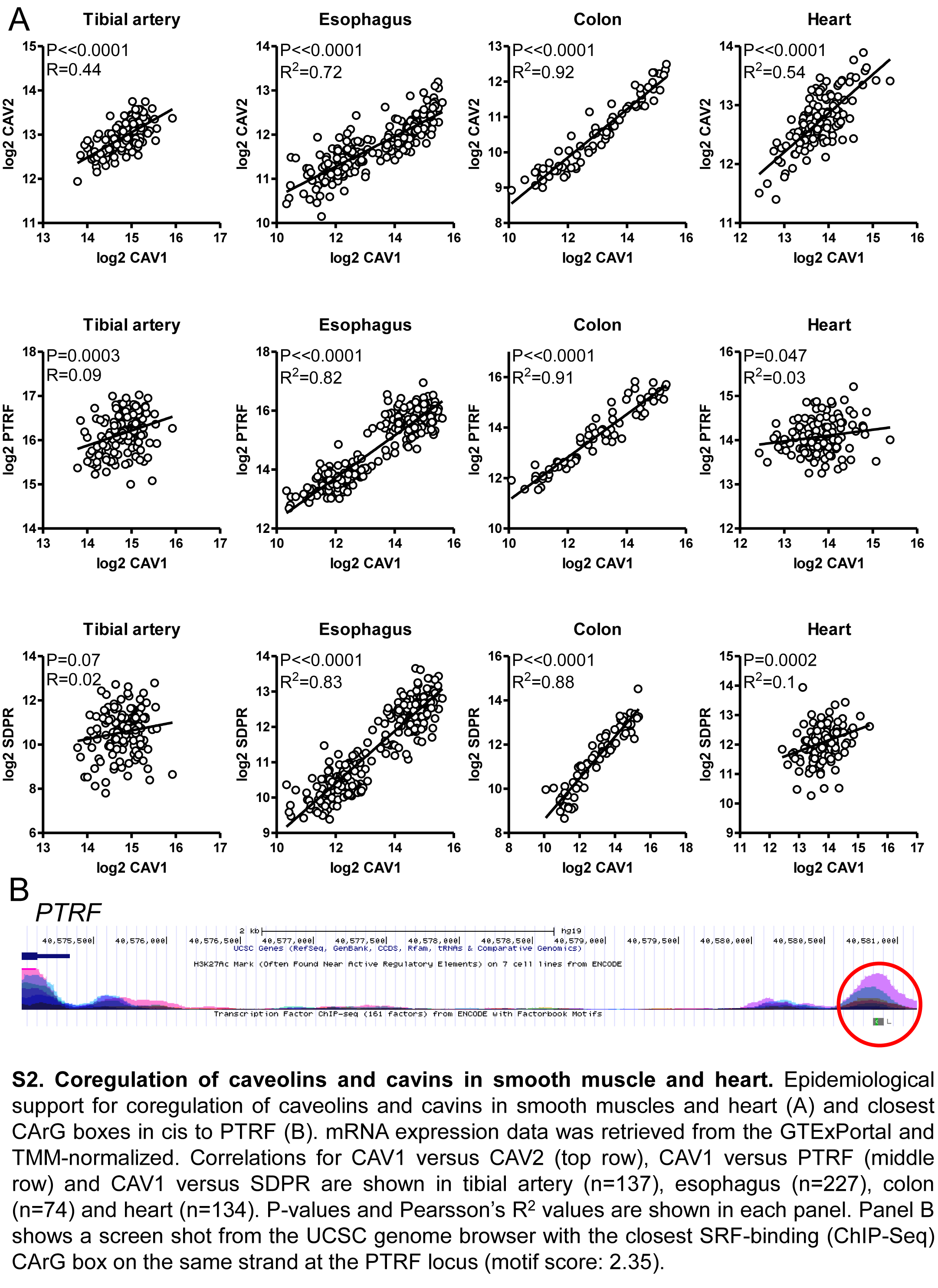

Supplement: S2 Fig — (TIF) [file pone.0133931.s002.tif]
